# *Rickettsia burneti* and *Brucella melitensis* co-infection: a case report and literature review

**DOI:** 10.1186/s12866-021-02323-x

**Published:** 2021-10-05

**Authors:** Jiangqin Song, Xiaorong Hu, Xiaolong Li, Youping Chen, Xiangyuan Yan, Weifang Zhu, Yan Ding, Junyang Zhou

**Affiliations:** 1 Laboratory Department, the First People’s Hospital of Tianmen City, Tianmen, 431700 Hubei China; 2 Gastrology department, the First People’s Hospital of Tianmen City, Tianmen, 431700 Hubei China; 3 General surgery department, the First People’s Hospital of Tianmen City, Tianmen, 431700 Hubei China; 4 Department of science and education, the First People’s Hospital of Tianmen City, Tianmen, 431700 Hubei China; 5 Hubei Key Laboratory of Embryonic Stem Cell Research, Hubei University of Medicine, Shiyan, 442000 Hubei China; 6 Department of Pathogen Biology and Immunology, Xuzhou Medical University, Xuzhou, 221004 Jiangsu China

**Keywords:** Rickettsia burneti, Brucella melitensis, brucellosis, Q-fever, coinfection

## Abstract

*Rickettsia* is the pathogen of *Q fever, Brucella ovis* is the pathogen of brucellosis, and both of them are Gram-negative bacteria which are parasitic in cells. The mixed infection of *rickettsia* and *Brucella ovis* is rarely reported in clinic. Early diagnosis and treatment are of great significance to the treatment and prognosis of *brucellosis* and *Q fever*. Here, we report a case of co-infection *Rickettsia burneti* and *Brucella melitensis*. The patient is a 49-year-old sheepherder, who was hospitalized with left forearm trauma. Three days after admission, the patient developed fever of 39.0°C, accompanied by sweating, fatigue, poor appetite and headache. Indirect immunofluorescence (IFA) was used to detect *Rickettsia burneti* IgM. After 72 hours of blood culture incubation, bacterial growth was detected in aerobic bottles, Gram-negative bacilli were found in culture medium smear, the colony was identified as *Brucella melitensis* by mass spectrometry. Patients were treated with doxycycline (100 mg bid, po) and rifampicin (600 mg qd, po) for 4 weeks. After treatment, the symptoms disappeared quickly, and there was no sign of recurrence or chronic infection. *Q fever* and *Brucella* may exist in high-risk practitioners, so we should routinely detect these two pathogens to prevent missed diagnosis.

## Background

*Brucella melitensis* is the pathogen of brucellosis, and *Rickettsia burneti* is the causative agent of Q fever, both of which are intracellular parasitic Gram-negative bacteria. Studies have shown that brucellosis and Q fever have roughly the same source of infection and route of transmission. *Brucella* can be transmitted from person to person, and vertical transmission is most common between mother and infant [1]. Brucellosis in humans can be severely debilitating, often with longterm adverse consequences for health [2]. Brucellosis in human beings will seriously weaken human body function, which usually causes long-term adverse consequences for health [3]. These two diseases can be prevalent in the same area at the same time, and the same patient (or animal) will be infected with these two diseases at the same time, which poses a great threat to human and animal health and public safety, and also causes great losses to the world economy. Up to now, few cases of mixed infection of *Brucella* and *rickettsia burgdorferi* have been reported [4, 5]. In PubMed, we found only one similar case report [6]. Brucellosis and Q fever are probably seriously underestimated and reported as lacking typical clinical symptoms, which are easy to be misdiagnosed and missed.

In this paper, the pathogenesis, clinical manifestations, laboratory examination results, diagnosis and treatment of a case of complicated infection were introduced in detail, and the related literatures were analyzed, in order to provide experience for the clinical treatment of this kind of disease.

## Case presentation

A male aged 49 years was admitted to the First People's Hospital of Tianmen City on May 18 2021 due to left forearm trauma. The patient had no history of surgical trauma, hypertension and diabetes or hepatitis. The patient is a shepherd. In the past three months, he has delivered sheep several times. He lives in Tianmen, Hubei Province, China, where there is no epidemic history of *Rickettsia* and *Brucella*.

**Fig. 1:**
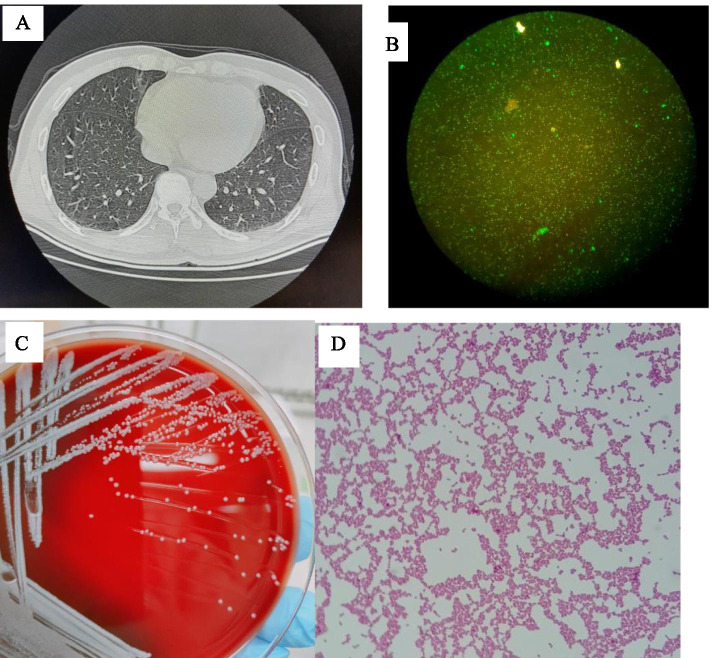
Chest CT and microbiological examination results during hospitalization. A. Chest CT showed a few chronic infective lesions in the lung. B. *Rickettsia Burneti* IgM was positive by IFA test. The positive bacteria emitted green fluorescence(200X). C. Images of pure bacteria after 48 hours of culture. Small, smooth and non-hemolytic colonies can be seen on the blood plate. D. Gram stain of *Brucella melitensis.* Gram-negative spherical microbacilli can be seen, blunt round at both ends, single or paired, short chain arrangement, no spore (400X)

The ulnar wound of the patient's left forearm is about 1cm, with severe contusion and slight bleeding. The left forearm is swollen and tender, with limited movement, and the left finger moves. X-ray showed fracture of the left ulna. Routine laboratory tests showed that there was no obvious abnormality in white blood cell (WBC), liver and kidney function, or coagulation function. Chest computed tomography (CT) showed some chronic infective lesions in the lungs (Fig. 1A). Emergency doctors gave debridement suture and plaster support for external fixation. On May 21, the patient developed fever, sweating, weakness, loss of appetite and headache, the highest body temperature was about 39.0°c, and the physical examination was normal. There is no obvious abnormality in blood routine (BRE), but procalcitonin (PCT) and C-reactive protein (CRP) are obviously increased (Table 1). After physical cooling treatment, the patient's fever was not significantly ameliorated. Blood culture test was carried out. Considering his direct contact with sheep, respiratory pathogens were detected by IFA, including Legionella pneumophila IgM*, Mycoplasma pneumoniae* IgM*, Rickettsia burgdorferi* IgM*, Chlamydia pneumoniae* IgM*, adenovirus* IgM*, respiratory syncytial virus* IgM*, influenza A virus* IgM*, influenza B virus* IgM *and parainfluenza virus* IgM*.* The IFA test was positive for *Rickettsia Burneti* IgM (Fig. 1B) and negative for all others. After 72 hours blood incubation, bacterial growth was detected in aerobic bottles, and the culture medium was extracted and inoculated in Columbia blood plate and chocolate plate without vancomycin, and placed in an incubator containing 5% CO _2_ at 35°C. Colonies appeared after 24 h of cultured. After 48 hours of culture, small, smooth and non-hemolytic colonies can be seen on the blood plate (Fig. 1C). Gram-negative spherical microbacterium can be seen from the extracted culture medium, with round ends, single chain or paired, short chain arrangement and no spores (Fig. 1D). The colony was identified as *Brucella melitensis* by mass spectrometry. Abdominal ultrasound showed no obvious lesions in liver and kidney. Electrocardiogram was normal.

**Table 1:** Laboratory tests results of the patient

	Reference range	May-19	May-23	May-28	Jun-4	Jun-11	Jun-28
WBC(×10^9^/L)	3.5-9.5	7.23	5.16	5.19	4.00	4.37	3.89
MON%(×10^9^/L)	3-10%	13.8	10.5	5.6	5.6	6.2	6.3
PLT(×10^9^/L )	125-350	194	218	243	243	224	194
PCT(pg/ml)	≤0.046	-	0.188	0.087	0.025	0.035	0.022
ALT(U/L)	0-40	53	-	44	153	78	37
AST(U/L)	0-40	41	-	28	69	52	23
CRP(mg/L)	<6	31.12-	31.62	23.34	3.19	4.37	1.72

White blood cell (WBC), monocyte count (MON#), platelet (PLT), procalcitonin (PCT), alanine aminotransferase (ALT), aspartate aminotransferase(AST), c-reactive protein (CRP), -: none.

According to the epidemiological investigation, the patient was a shepherd who raised sheep for more than one year. The patient gave birth to sheep many times in the past three months, but he did not take any anti-infection measures. He did not eat fresh lamb or have goat milk. In view of the clinical feature, laboratory test and epidemiological history (contact with sheep), we suspected the patient had brucellosis and Q-fever. Patient received targeted antimicrobial therapy: doxycycline (100 mg bid, po) and rifampicin (600 mg qd, po). After three days of treatment, the patient's temperature returned to normal, and symptoms such as fatigue were obviously improved. The patient was then discharged from hospital and continued to receive the same treatment at home. However, on June 4th, the patient's liver function indexes, alanine aminotransferase (ALT) and aspartate aminotransferase (AST), were significantly elevated (Table 1). Considering the side effects caused by taking doxycycline and rifampicin, the patient received additional treatment (Glutathione Buccal Tablets, 0.1g tid, po). During the follow-up, the patient's liver function gradually returned to normal (Table 1). The patient is now in good condition, showing no signs of recurrence or chronic infection.

## Discussion

*Rickettsia burneti* is an intracellular parasitic Gram-negative bacterium, which can cause human acute Q fever. Human infection may occur due to inhalation of dust contaminated by body fluids of infected animals, consumption of unpasteurized dairy products and contact with milk, urine, feces, vaginal mucus or semen of infected animals [7]. The people with the highest risk of this infection are farmers, laboratory workers, sheep and dairy workers and veterinarians [8]. Q fever sufferers may have high fever, chills, severe headache and muscle aches. A few patients may have sore throat, nausea, vomiting, diarrhea, abdominal pain and delirium. The clinical manifestations of Q fever were non-specific and 60% asymptoms, which may cause a large number of misdiagnosis and missed diagnosis. Hepatitis, endocarditis or meningitis are complications observed in rare chronic course disease, with a mortality rate of up to 65%. If left untreated, the mortality rate of acute forms is as high as 1-2 %[9]. Q fever is effective in the early treatment of the disease with antibiotics. If the treatment is delayed, it will easily lead to chronic Q fever, with long treatment period, easy recurrence and high mortality. Therefore, early and accurate diagnosis of Q fever is extremely important. At present, the diagnosis of Q fever mainly relies on serological and molecular biological methods. However, *Rickettsia* infection is often ignored by people, and the bacteremia period is very short. In addition, *Rickettsia* is an intracellular parasitic bacterium, and the bacteria content in clinical samples is very low, which makes it difficult to detection. In our case, the patient developed symptoms such as high fever, excessive sweating, weakness, loss of appetite and headache, but no rash was found. WBC, platelet (PLT), ALT, AST showed no obvious changes, but PCT and CRP increased significantly (Table 1). Chest CT showed no obvious abnormality (Fig. 1A). In addition, by IFA test, *Rickettsia Burneti* IgM was positive in patients' serum (Fig. 1B). Although the patient's clinical symptoms were atypical and there was no obvious abnormality in laboratory examination or CT, it is suspected to be Q fever considering the epidemiological history (contact with sheep).

Brucellosis is a neglected bacterial zoonotic disease, which seriously weakens people's health and usually causes long-term adverse consequences for health [2]. Brucellosis is caused by genus *Brucella* bacteria. The species considered as important vectors of human disease are B. Melitensis, B. Abortus and B.Sui s[2]. *Brucella* is more common in cattle, sheep, pigs and other domestic animals. Patients are mainly infected by contacting infected sheep or by drinking infected goat milk*. Brucella* can also be transmitted from person to person, and the most common is vertical transmission from mother to child [12]. The incubation period is usually 5-60 days [13]. After infection, the symptoms of Brucellosis are atypical and the clinical manifestations are varied, including fever, bone and arthropathy, sweating, fatigue, etc., which can be combined with other diseases. Because of these characteristics, it is easy to cause clinical missed diagnosis and misdiagnosis, delay the disease, and even cause complications such as arthritis, myocarditis and liver and spleen involvement [13]. Many studies have shown that CRP and PCT are sensitive indicators for the diagnosis of Brucellosis [17, 18]. The cure rate of acute brucellosis infection is 90-95%, while the chronic infection caused by brucellosis has not been cured, so early diagnosis and treatment of brucellosis are very important. After 72 hours of blood culture, the culture system reported positive results. Gram-negative spherical bacilli were found in the extracted culture medium (Fig. 1C). After 24 hours of pure bacteria culture, tiny colonies grew on the plate, after 48 hours of culture, typical *Brucella* colony morphology appeared on the plate (Fig. 1D). The colony was identified as *Brucella melitensis* by mass spectrometry, so we speculate that the patient was also infected with *Brucella.* In laboratory tests, we noticed a significantly increase in PCT and CRP (Table 1).

Brucellosis and Q fever are zoonotic infectious diseases that attracted worldwide attention. They have the same infection source, host, transmission route and have similar clinical manifestations. The incidence of interstitial pneumonitis and bronchopneumonia in *Brucella* infections is low [19, 20] , However, almost half of patients with acute *rickettsia burgdorferi* infection will develop pneumonia [21]. The incidence of Q fever is lower, but its organ damage is more serious and its mortality is higher. If these two diseases enter the chronic phase, it will be difficult to cure them, so early diagnosis and symptomatic treatment are very important. The mixed infection of *Rickettsia burneti* and *Brucella melitensis* is rarely reported in clinical. As far as we know, this is the first case report in China. In PubMed, we only found one similar case report [6]. Although it is rare for patients to be infected with *Brucella* and *Rickettsia* at the same time, we should not ignore this situation. For potential infected persons, we should adopt a variety of detection methods to improve the detection rate of the pathogen. Since brucellosis and Q-fever have the same sensitive drugs [22, 23], patients were treated with doxycycline and rifampicin. Three days later, the patient's temperature returned to normal. After the patient was discharged from hospital, the treatment plan continued. Ten days later, the patient's liver function became abnormal (Table 1). Because doxycycline and rifampicin may cause liver damage, so we added liver protection drugs. During the latest follow-up, the patient's liver function returned to normal (Table 1). Therefore, we should pay attention to the detection of changes in liver function when treating co-infection.

In summary, we report a case of co-infection of *Rickettsia burneti* and *Brucella melitensis*. The patient was hospitalized for fracture, and developed high fever three days after admission, with no any other obvious discomfort. Laboratory examination showed that PCT and CRP were elevated, and there was no any other obvious abnormality. Through IFA and mass spectrometry, we confirmed that the patient was infected with *Rickettsia burneti* and *Brucella melitensis*. After active treatment with doxycycline and rifampicin, the patient's condition improved significantly. For high-risk practitioners, Q fever and brucellosis may exist in one patient. We should routinely detect these two pathogens through a various tests to prevent missed diagnosis. In the follow-up treatment, we should pay attention to the side effects of drugs, and actively use liver protective drugs to prevent liver function damage.

## Data Availability

All data y are included in this article

## Abbreviations

IFA: indirect immunofluorescence assay
WBC: white blood cell
CT: computed tomography
BRE: Blood routine examination
CRP: C-reactive protein
ALT: alanine aminotransferase
AST: aspartate aminotransferase
